# Dataset on energy consumption in buildings within tropical climate based on design aspects of courtyards

**DOI:** 10.1016/j.dib.2025.111834

**Published:** 2025-06-24

**Authors:** Abdulbasit Almhafdy, Ashjan Al-Mutairi, Asma Al-Shargabi, Amal Al-Shargabi

**Affiliations:** a Department of Architecture, College of Architecture and Planning, Qassim University, Buraydah, Saudi Arabia; b Independent Researcher, Riyadh 11461, Saudi Arabia; c Department of Information Technology, College of Computer, Qassim University, Buraydah, 51452, Saudi Arabia; d Institute of Artificial Intelligence, School of Computer Science and Informatics, De Montfort University, Leicester, LE1 9BH, United Kingdom

**Keywords:** Energy consumption, Sustainable architectural design, Optimization, Courtyard design, Smart cities, Big data

## Abstract

Sustainability and energy efficiency have become fundamental objectives for modern society. Green roofs and facades are increasingly recognized as innovative and sustainable strategies to improve the energy performance of buildings. This paper introduces a dataset about buildings thermal performance and energy consumption in tropical climate depending on adjacent outdoor enclosed courtyards design features with different architectural shapes U, L, and O. The core data has been collected in public building in Kuala Lumpur, Malaysia. Then it expanded using simulation. The core measured raw data is the temperature and the other data is simulated and/or calculated. The dataset includes detailed design features of courtyards such as plan aspect ratio, number of floors, and orientation. Measurement instruments were calibrated against real-world measurements to ensure accuracy and reliability. The simulated data is tested and validated based on the statistical aspects of the raw data using Pearson correlation coefficient, with a value of 0.882. The dataset includes total 8,685 records across the different courtyard' shapes. This dataset captures intricate relationships between architectural design parameters and energy consumption, making it a valuable resource for architects, engineers, and researchers interested in optimizing building designs for improved energy efficiency. It also allows in-depth analysis and potential reuse in studies related to sustainable architecture and urban planning.

Specifications Table

**Table:** 

Subject	Sustainable Urban Design
Specific subject area	The effect of design aspect of outdoor enclosed courtyards on energy consumption of adjacent buildings.
Data format	Raw, Analysed Raw data is measured in field using instruments and then the analysed/simulated data is created using IESVE simulator. It is then tested and validated using Pearson correlation coefficient, with a value of 0.882
Type of data	Table
Data collection	Real-world data were gathered using a Heat Stress Wet Bulb Globe Temperature (WBGT) Meter to measure temperature, humidity, and mean radiant temperature. This instrument measured the data of the buildings adjacent outdoor enclosed courtyards. The cite is a public building in Kuala Lumpur, Malaysia. This collected data is used as a seed to generate more data using IESVE simulation. The generated data is statistically tested and verified.
Data source location	Institution: A Public Building City/Town/Region: Kuala Lumpur Country: Malaysia
Data accessibility	Repository name: DatasetOfEnergyWithDifferentCourtyardsShapes-EDCS Data identification number: doi:10.5281/zenodo.14626639 Direct URL to data: https://doi.org/10.5281/zenodo.15073304

## 1 Value of the Data


 

• This dataset provides insights into the effect of different architectural courtyard forms under different design conditions on the buildings thermal and energy performance. particularly, understanding how the outdoor enclosed courtyards with different architectural shapes—U, L, and O—affect the energy performance of adjacent buildings in tropical climates. Buildings in these regions face unique challenges in energy consumption due to high temperatures and humidity levels.
• This dataset can be used to model and simulate energy performance in similar or modified architectural designs. The data can also be employed in comparative studies to evaluate the effectiveness of different design strategies in various climatic conditions.
• This dataset can serve as an educational tool in architecture and engineering courses, helping students understand the implicit relationship between building/courtyards design, and energy efficiency. It can be used for practical exercises in simulation and modelling software, providing hands-on experience in optimizing building/courtyards designs.

## 2 Background

Sustainability and energy efficiency have become fundamental objectives for modern society. For example: green roofs and courtyards are increasingly recognized as innovative and sustainable strategies to improve the energy performance of buildings [1]. Designing buildings based on specific climatic characteristics plays a crucial role in creating comfortable indoor environments and conserving energy [2]. Incorporating a courtyard as part of the building's external structure can significantly reduce cooling demands by facilitating ample airflow. Computer technology and building energy simulation tools have been integrated with various methods to evaluate the cost-effectiveness of energy conservation and efficiency measures [[3], [4], [5], [6], [7], [8], [9], [10], [11], [12], [13], [14], [15], [16], [17]]. A number of scientific studies have focused on generating simulated datasets tailored for urban and building-scale design [[5], [6], [7], [8], [9], [10], [11], [12], [13], [14], [15], [16], [17]]. These datasets serve as valuable resources for analysis, modeling, and decision-making. For instance, the ReCo dataset [9] offers the largest open-source collection of residential community layouts, synthetically generated using generative adversarial networks (GANs) to analyze urban morphological patterns. Similarly, UrbanScene3D [10] provides synthetic 3D urban environments created with Unreal Engine and LiDAR data, enabling high-fidelity scene understanding and analysis. The HiTAB dataset [11] delivers detailed mappings of tree and building heights in Chicago at meter-level resolution, aiding microclimate and morphology modeling. SpaceNet 7 [12] offers spatiotemporal building footprint data for over 100 global cities to simulate urban development dynamics over time. Additionally. Building3D [13] provides extensive 3D point clouds and mesh data for over 160,000 buildings across 16 cities, supporting AI-based reconstruction of building geometry. Jylhä et al. [14] presented a study that uses synthetic future hourly weather data—generated via climate model outputs and morphing techniques—to simulate heating and cooling demand using the IDA ICE simulation tool. Ali et al. [15] developed a dataset for one million synthetic residential buildings in Dublin using EnergyPlus and DesignBuilder to simulate energy performance across diverse typologies. Authors in [16] contributed a dataset covering energy renovation projects in Latvia, where some performance data was generated using simulation methods to assess energy improvements. Lastly, Emami et al. [17] introduced BuildingsBench, a large-scale synthetic dataset simulating energy consumption patterns for 900,000 U.S. buildings, designed to support short-term energy load forecasting. Collectively, these studies highlight the critical role of simulated data in advancing research and planning for sustainable, energy-efficient, and resilient urban environments.

This paper provides a data on the thermal performance and energy consumption of buildings adjacent to outdoor enclosed courtyards with different architectural shapes (L, U, O). This study is an experimental study and the approach involves a combination of parametric modeling of the design environment using IESVE simulation software as well as real data collection; this data has been collected in Kuala Lumpur, Malaysia, then, it is used as seeds to generate the whole data. Then validity of generated data is statistically tested and verified. The availability of this data and similar ones allows researchers, architects, and construction companies to analyze the impact of architectural design factors such as orientation and shape - as in this data - and the proportions of windows in building facades and roof configurations on energy efficiency. This data can contribute to improve the quality of architectural design based on accurate scientific analysis. This data also allows exploring the implicit relationship between design and environmental factors on the operational efficiency of buildings and constructions.

The simulation process was adopted to generate data based on real measurements taken over a week as clarified in the previous section. Then, the statistical features of the generated data are compared with those of the actual data to validate their accuracy which is exactly what was done in this study.

## 3 Data Description

This dataset was collected from a public building in Kuala Lumpur, Malaysia, as an example of tropical climate area. This building is adjacent to different outdoor enclosed courtyards. The data was gathered over one week with energy consumption measured every 5-15 minutes. This measured data was then used as a seed to generate the whole dataset using simulation. The simulation process is configured by the tropical climate data conditions and also then tested and verified based on the statistics features of the real measured data. This data was gathered as a part of one author of this paper PhD thesis [4].

The dataset includes 3 Excel files, each file represents the data of courtyards and also the data of the energy consumption in the adjacent building. Therefore, each file provides two major data dimensions, the first one is the input data and the other is the output data. The input data includes the design variables such as the courtyards 's width, length, height, orientation (North, South, East, West), and window ratios, which are essential for determining the architectural layout. For the output columns, the dataset includes cooling load, heating load, and total energy load. The 3 files are briefly described as follow; the number of columns is different based on the nature of the architecture aspects of the courtyard shape:

1) **L_SHAPE_DATA.xlsx**
  Contains 10 columns and 1537 records.
2) **O_SHAPE_DATA.xlsx**
  Contains 10 columns and 5611 records.
3) **U_SHAPE_DATA.xlsx**
  Contains 10 columns and 1537 records.

The following tables provide a detailed description of the variables and their respective ranges across all shapes, along with additional shape-specific variable for U-shaped and L -shaped courtyards.

**Shared Variables Across All Shapes (L, O, and U)**

**Table:** 

Variable	Description	Range

WIDTH (m)	The width of the courtyard in meters.	30-60
LENGTH (m)	The length of the courtyard in meters.	30 - 60
HIGHT (m)	The height of the adjacent building in floors.	8-16
ORIENTATION (°)	The orientation of the courtyard, measured in degrees.	0-315
WINDOWS_RATIO (%)	Ratio of windows to total wall area.	0.1-0.4
FORM_FACTOR	A shape factor of courtyard that influences energy performance.	0.8 - 1.89
S/V_RATIO (%)	Surface-to-volume ratio, a key factor in determining energy performance.	0.19 - 0.47
COOLING_LOAD (KWH/m²)	Cooling load required for the building.	43.0 - 57.1
HEATING_LOAD (KWH/m²)	Heating load required for the building.	0.58 - 2.65
TOTAL_LOAD (KWH/m²)	Total energy load required for the building (cooling + heating)	45-59

**Fig. 1:**
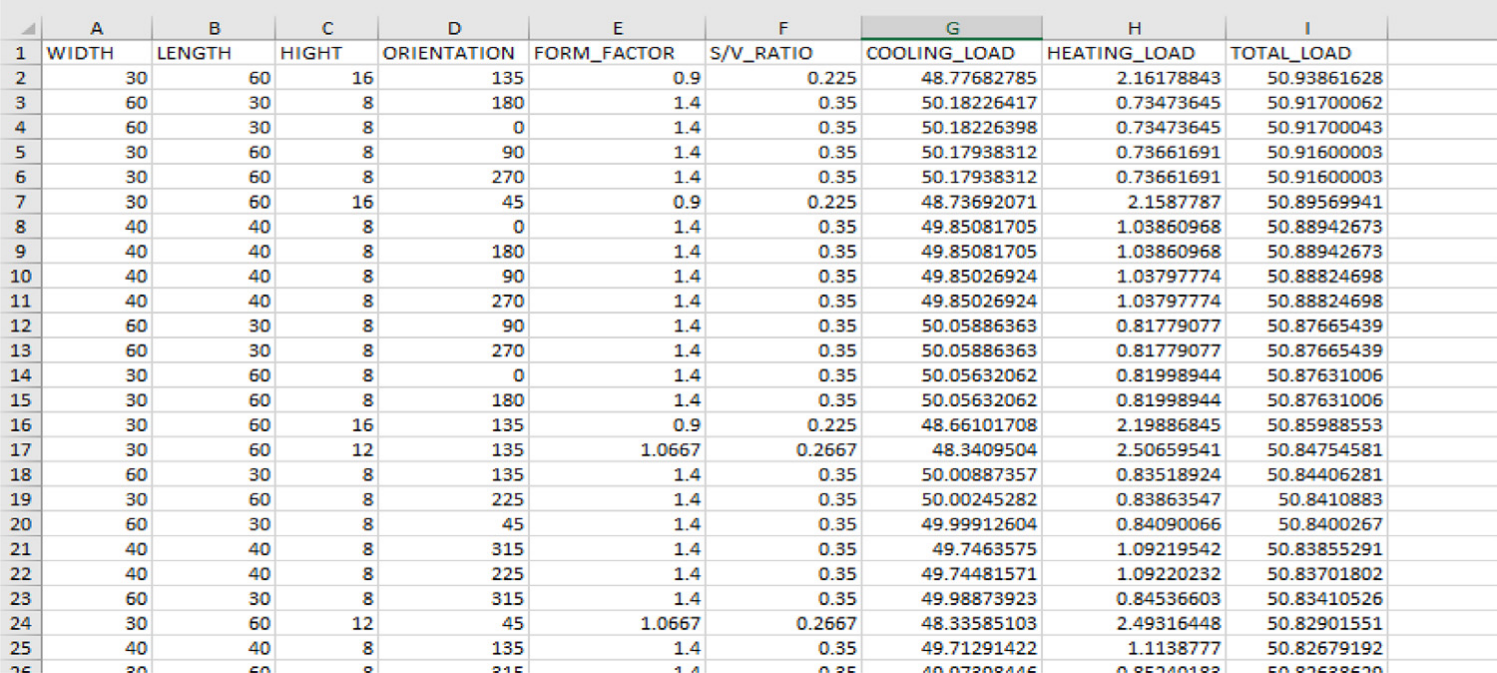
A sample of data of for O_SHAPE file

Fig. 1 shows a sample of data of **O_SHAPE_DATA.xlsx**.

## 4 Experimental Design, Materials and Methods

This section describes the experimental methods used to collect, generate and validate the dataset, focusing on the energy performance of UEC courtyards. The study was conducted using simulation modeling with IESVE and validated through field measurements with a Heat Stress WBGT Meter. The correlation between simulated and measured data confirms the validity of the simulation model, making the dataset reliable for further analysis. Below is a description of the experimental approach, tools, and methods used to acquire the dataset.

The field measurements were conducted in buildings with various architectural shapes located in a public building in Kuala Lumpur, as a representative case of buildings in tropical climate.

### 4.1 Data collection process

This section describes the process that produces the dataset. Basically, there was 3 stages where the basic core data is measured in field and then we use simulation to get the whole data, finally we test the validity of the simulated data. The main core raw data is the temperature, and the other data are simulated and/or calculated. The main stages can be described in details as follow:

### 4.2 Field measurements

To validate the simulation results, real-world measurements were conducted using a Heat Stress Wet Bulb Globe Temperature (WBGT) Meter (Fig. 3). The Extech HT30 Heat Stress WBGT Meter was selected for this study due to its proven accuracy and reliability in measuring ambient temperature and thermal comfort conditions. This instrument measures temperature (TA), relative humidity (RH), and Mean Radiant Temperature (MRT), which indicates the effects of direct solar radiation on surfaces.

• **Calibration and Instrument Testing**

Prior to commencing field measurements, the device was calibrated and tested against two additional weather stations (w-station2 and w-station3) to verify the consistency and validity of its readings.

**Fig. 2:**
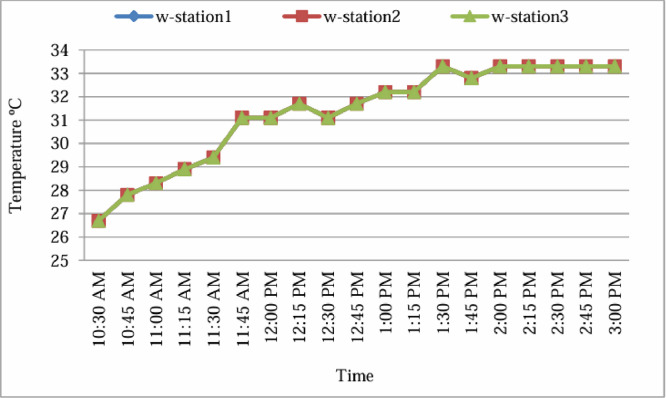
Instrument test before using.

As shown in Fig. 2, temperature data were recorded simultaneously by all three instruments over a calibration period from 10:30 AM to 3:00 PM. The recorded measurements followed a nearly identical trend, with a maximum variation not exceeding 0.2°C throughout the period. This strong agreement confirms the high precision and reliability of the Extech HT30 device.

**Fig. 3:**
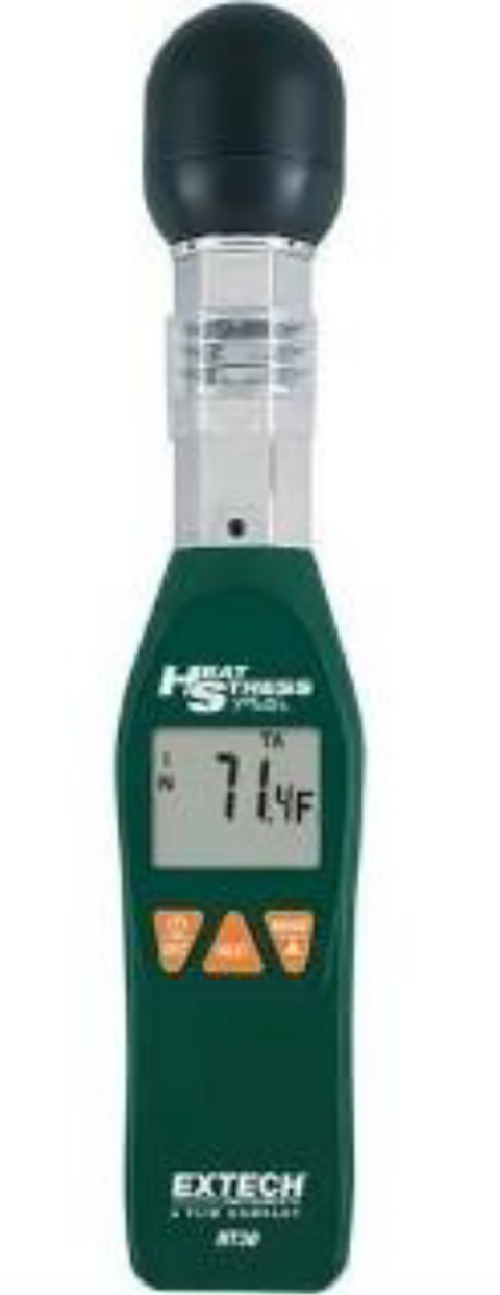
The Extech HT30 heat stress WBGT meter used for data collection.

**Fig. 4:**
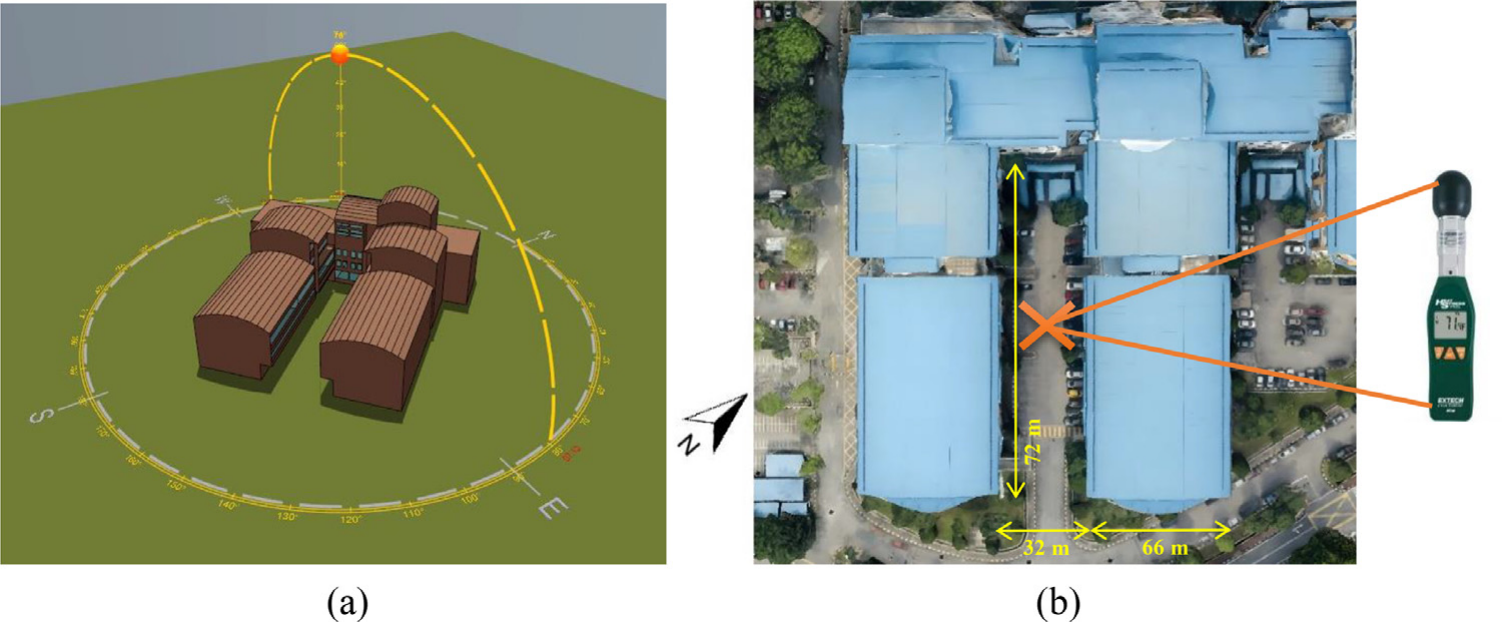
(a)The UEC U_Shape courtyard in model viewer (IESVE) [4], (b) Google image of the UEC at the UiTM civil engineering facility.

This calibration step was essential to ensure that neither environmental variation nor instrumental error would influence the accuracy of the main data collection. To maintain consistency during the actual field study, temperature data were collected at the centre of the courtyard, positioned 1.5 meters above the ground level (see Fig. 4-b), which corresponds to the average eye level of an adult.

The specification of the use instrument is clarified by the following table.

**Extech HT30 heat stress WBGT meter technical specifications**

**Table:** 

Specifications	Range	Basic Accuracy
Wet Bulb Globe Temperature (WBGT)	32 to 122°F (0 to 50°C)	±4°F/2°C
Black Globe Temperature (TG)	32 to 176°F (0 to 80°C)	±4°F/2°C
Air Temperature (TA)	32 to 122°°F (0 to 50°C)	±1.8°F/1.0°C
Humidity	0 to 100%RH	±3%RH

Fig. 4-a shows a model of U-Shape courtyard using IESVE viewer. Measurements were taken over one week, with data recorded at hourly intervals from August 20 to August 26. The experimental conditions are as follows:

• ***Outdoor environment***: The outdoor temperature during the data collection period ranged from 28°C to 34°C, and the relative humidity levels were between 70% and 90%.
• ***Indoor environment****:* For the purposes of the simulation, the indoor areas adjacent to the courtyard were maintained at a constant temperature of 22°C, representing fully air-conditioned spaces. This allowed the energy loads to be directly attributable to the architectural design rather than indoor climate variations. The selection of a constant indoor air temperature of 22 °C is based on the" CIBSE Guide A" recommendation of 21–23 °C as a thermally comfortable range for office environments [18]. This value represents a reasonable midpoint within the recommended range and is commonly used in building simulations to reflect stable and comfortable indoor conditions. In the context of Malaysia’s hot and humid climate, 22 °C is also a typical setpoint in government and office buildings, making it a practical and contextually appropriate choice. "CIBSE Guide A" is a widely recognised reference published by the Chartered Institution of Building Services Engineers. It provides comprehensive guidance on environmental design criteria, including thermal comfort, ventilation, and indoor temperature ranges for various building types, with a particular focus on energy-efficient and occupant-friendly design [18].

### 4.3 Simulation process

This section is devoted to describe the simulation process, conditions, and validation. The simulation process was adopted to generate data based on real measurements taken over a week as clarified in the previous section. This method is widely used in many studies when it is difficult to measure all data directly [[5], [6], [7], [8]]. Instead, a subset of the data is measured, and the rest is generated through simulation to ensure the accuracy of the process. Then, the statistical features of the generated data are compared with those of the actual data to validate their accuracy which is exactly what was done in this study. The accuracy of the data and the consistency of its statistical features were tested using Pearson’s method, as will be explained in the following section. The simulation model was created using IESVE (Integrated Environmental Solutions Virtual Environment), a software tool designed for simulating and analyzing building energy performance. This study utilized the Integrated Environmental Solution IESVE software to conduct the parametric analysis of environmental performance. The IES Virtual Environment has been thoroughly evaluated and validated in accordance with various global and regional standards. Some examples of these standards include:

• ASHRAE 140: 2007 Reports (VE 2014)
• US Green Building Council (USGBC)
• Chartered Institution of Building Services Engineers (CIBSE)
• UK National Calculation Methodology
• ASHRAE 55 calculation procedure
• ASHRAE 62.1 calculation procedure
• California Energy Commission
• US Department of Energy
• ISO 7730 calculation procedure
• South Africa National Building Regulations

The energy performance of the building was calculated/analyzed. The indoor environment was modelled as fully air-conditioned at a constant temperature of 22°C. Energy loads were calculated in megawatt-hours per square meter (MWh/m²) for heating, cooling, and total energy consumption. Results were normalized for comparison.

The simulation results from IESVE were compared with the field measurements. The simulation data were recorded, and the Mean Radiant Temperature (MRT) values were matched with the measured field data to ensure the consistency.

### 4.4 Data analysis and processing

This section describes the type of analysis that is applied on data. At first, the results from the IESVE simulations and field measurements were normalized for consistency, allowing accurate comparison across different design scenarios. Then both simulation and field measurement data were analyzed to assess/calculate the energy performance of various courtyards design factors. The outlier data was detected and eliminated. The energy performance of the attached built volume was evaluated by investigating the cooling, heating, and total energy consumption (expressed in megawatt-hours per square meter, MWh/m²) for each scenario.

**Fig. 5:**
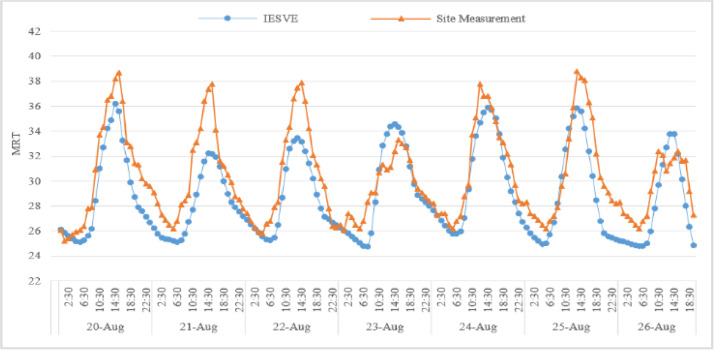
MRT obtained through both field measurement and simulation.

Finally, the correlation between the simulation results and field measurements was calculated using Pearson correlation coefficient, with a value of 0.882, indicating a strong correlation between the simulated and measured data. Fig. 5 reveals the multiple range test MRT obtained through both measurement and simulation. Obviously, the data shows high level of correlation between filed measurement and simulation data. The correlation between the simulation and field data was strong, with a Pearson correlation coefficient of 0.882, confirming the accuracy of the simulation model as shown by Fig. 5. The deviation observed on August 21st can be attributed to various real-life factors that are not fully captured by the simulation model. Nevertheless, the accuracy of the model was evaluated using the Pearson correlation coefficient between simulated and measured temperatures, which yielded a value of 0.882, indicating a strong positive correlation that supports the model’s validity for analytical and comparative purposes. Although the peak difference of 6 °C is notable, the overall alignment in trend and statistical correlation demonstrates that the model is sufficiently reliable for energy analysis. Validation metrics were applied in accordance with ASHRAE Guideline 14 (2014), which recommends using statistical indicators such as R², NMBE, and CV(RMSE) to ensure consistency between simulated and measured data [19].

This correlation supports the reliability of the IESVE simulator for further studies on courtyard design.

## Limitations

This dataset is specifically focused on buildings located in tropical climates, which may limit its applicability to regions with different or variable weather conditions. The uniformity of the tropical climate throughout the year restricts the dataset’s ability to capture seasonal variations in energy load patterns.

The data collection process was constrained to a period of seven consecutive days due to limited access to the facilities. Furthermore, the recording interval was set on an hourly basis, determined by the technical specifications and capabilities of the devices and sensors utilized.

Additionally, the scope of the dataset is primarily confined to the assessment of energy load. Other critical factors such as cost considerations, material sustainability, and occupant comfort were not included in the dataset, which may affect the comprehensiveness of the findings.

## Ethics statement

The authors have read and follow the ethical requirements for publication in Data in Brief and confirming that the current work does not involve human subjects, animal experiments, or any data collected from social media platforms.

## Credit author statement

This research was conducted through a joint effort between the group of authors as follows: the field study – by Abdulbasit Almhfadi, data processing and preliminary writing by Ashjan Al-Mutairi, and Asma Al-Shargabi, verification, review and proof reading by Amal Al_Shargabi.

## Data Availability

GithubCourtyards Architecture Aspects with energy consumption (Original data)
